# The dual function of mitophagy in ferroptosis

**DOI:** 10.52601/bpr.2025.240071

**Published:** 2026-04-30

**Authors:** Xiaoxuan Zeng, Xushan Ma, Yueping Bai, Jiaqi Li, Fan Yu

**Affiliations:** 1 State Key Laboratory of Medicinal Chemical Biology, College of Pharmacy, Key Laboratory of Molecular Drug Research and KLMDASR of Tianjin, Nankai University, Tianjin 300350, China; 2 School of Health and Life Sciences, Qingdao Central Hospital, University of Health and Rehabilitation Sciences, Qingdao 266113, Shandong, China

**Keywords:** Mitophagy, Ferroptosis, ROS, Iron

## Abstract

Ferroptosis is a new form of cell death driven by iron-dependent lipid peroxidation. Thus, it is closely related to the lipid and iron metabolism. Accumulating evidence has suggested mitochondria, the center of cell metabolism, are important regulators of ferroptosis. This is not surprising as mitochondria are also the center for lipid metabolism and iron metabolism, as well as redox balance. As the essential way of mitochondrial quality control, mitophagy may alleviate ferroptosis. On the other hand, the digestion of iron-rich mitochondria may provide ample sources for the activation of ferroptosis. This review describes these new findings about the interplay of mitophagy and ferroptosis and demonstrates the dual role of mitophagy in ferroptosis.

## INTRODUCTION

Ferroptosis is an emerging form of regulated necrosis that arises from the accumulation of iron-dependent lipid peroxides. This cell death process is triggered by disruptions in cellular redox balance, which can occur through two primary mechanisms: either by inhibiting the uptake of cysteine, an essential amino acid required for the synthesis of cellular glutathione (GSH), or by inactivating the enzyme glutathione peroxidase 4 (GPX4), which is crucial for the detoxification of phospholipid hydroperoxides (Bi *et al.* 2024; Wei 2024a). When these defenses are compromised, lipid peroxidation escalates, leading to the characteristic cell death known as ferroptosis (Stockwell 2022). Mitochondria, often referred to as the cellular powerhouses, play pivotal roles in regulating iron metabolism, redox balance, and lipid metabolism. Disruptions in these processes impose stress on mitochondria, thereby altering their metabolism and behavior. Mild stress can enhance mitochondrial fission and activate mitophagy, a selective cellular process for the removal of damaged or superfluous mitochondria (Paing *et al.* 2024). In contrast, severe stress can induce cellular senescence and even programmed cell death. It is well established that mitochondria are central to the control of apoptosis, a distinct form of cell death. The Bcl-2 family of proteins and their regulation of outer membrane permeabilization for cytochrome c release, which subsequently activates the caspase cascade, are key components of this process (Qi *et al.* 2023). Currently, in the realm of disease research, the fields of mitophagy and ferroptosis have been the subject of in-depth investigation. Specifically, an over-activation of mitophagy has been found to be intricately linked to the progression of cardiovascular diseases. Moreover, mitophagy has been demonstrated to have a close association with traumatic brain injury, while ferroptosis shows a strong connection to ischemic stroke (Guo *et al.* 2023; Luan *et al.* 2023; Maremonti *et al.* 2024; Wei *et al.* 2024b; Zhang *et al.* 2024b).

Recently, accumulating evidence has suggested that mitochondria play a critical role in ferroptosis (Oh *et al.* 2022). Perturbations in mitochondrial metabolism and homeostasis contribute to ferroptotic sensitivity, involving aspects such as mitochondrial iron homeostasis, lipid metabolism, glutamine metabolism, and other metabolic pathways. A hallmark of ferroptosis is the distinctive morphological changes in mitochondria, such as shrinkage, increased membrane density, and enhanced mitochondrial fragmentation (Oh *et al.* 2022). These mitochondrial alterations are specific to the treatment with ferroptosis inducers and are not observed with other cell death inducers. As a result, these mitochondrial characteristics are considered one of the defining features of ferroptosis. However, the precise role of mitochondria in ferroptosis remains a subject of debate. While there is evidence suggesting that mitochondrial morphology changes are unique to ferroptosis, the functional implications and the mechanistic links between these changes and the execution of ferroptosis are not yet fully understood, highlighting the need for further research in this area (Xiao and Brown 2022).

Mitophagy is a vital cellular process that selectively targets damaged or superfluous mitochondria for degradation through the autolysosomal pathway (Chen *et al.* 2024). It is crucial for mitochondrial quality control, metabolic reprogramming, and the maintenance of mitochondrial homeostasis, and it plays a role in the prevention of cell death. However, the interplay between ferroptosis and mitophagy remains elusive. Emerging research suggests that mitophagy can either prevent or promote ferroptosis, depending on cellular or environmental cues (Yu *et al.* 2022). Accumulating evidence indicates that mitochondria are a key factor in ferroptosis, with perturbations in mitochondrial iron homeostasis, lipid metabolism, glutamine metabolism, and other metabolic pathways contributing to ferroptotic sensitivity. These changes also affect mitochondrial behavior, including mitophagy. The precise role of mitophagy in ferroptosis is not yet clearly defined. Here we will review recent advances and focus on the dual role of mitophagy in ferroptosis.

## MITOPHAGY

As the central hub for cellular energy metabolism, mitochondria are actively involved in a spectrum of vital metabolic processes, including adenosine triphosphate (ATP) synthesis, calcium ion buffering, programmed cell death (apoptosis), and fatty acid degradation (Xiao and Brown 2022). The preservation of mitochondrial quality and quantity is essential for the normal functioning of cells and tissues. Under conditions of excessive reactive oxygen species (ROS) production, such as during periods of starvation, mitochondria can incur significant damage (Zorov *et al.* 2014). To counteract this, mitochondria engage in a selective autophagy process, termed mitophagy, which facilitates the removal of damaged or superfluous mitochondria, thereby contributing to the maintenance of mitochondrial homeostasis.

Mitophagy, a selective form of autophagy, can be categorized into two primary mechanisms based on the initiating factors and the mitophagosome formation process: the PINK1-Parkin mediated pathway and the ubiquitin-independent pathway (Onishi *et al.* 2021; Youle and Narendra 2011). In the PINK1-Parkin pathway, PINK1 (phosphatase and tensin homolog-induced kinase 1) is instrumental in monitoring mitochondrial integrity. Under physiological conditions, PINK1 is translocated to the inner mitochondrial membrane and subsequently degraded. However, in mitochondria with compromised membrane potential, PINK1 accumulates on the outer mitochondrial membrane, initiating mitophagy through a cascade of reactions. Upon being activated, PINK1 initiates the phosphorylation process on Parkin, which functions as an E3 ubiquitin ligase. Subsequently, the phosphorylated Parkin is recruited to the outer mitochondrial membrane, where it plays a catalytic role in facilitating the ubiquitination of proteins located on the outer mitochondrial membrane. These ubiquitinated proteins are recognized by mitophagy cargo receptors, such as Optineurin (OPTN) and CALCOCO2/Nuclear dot protein 52 (NDP52) (Kataura *et al.* 2023), which possess a Ubiquitin Binding Domain (UBD) for interaction with ubiquitin. Additionally, these receptors contain an LC3-Interacting Region (LIR) motif, enabling them to bind Autophagy-related protein 8 (Atg8) and Microtubule-associated proteins 1A/1B light chain 3B (LC3), which are essential for the elongation of the phagophore membrane and the sequestration of mitochondria into autophagosomes. This process is critical for the maintenance of mitochondrial homeostasis and the removal of damaged mitochondria, thereby preserving cellular health and function.

**Figure 1:**
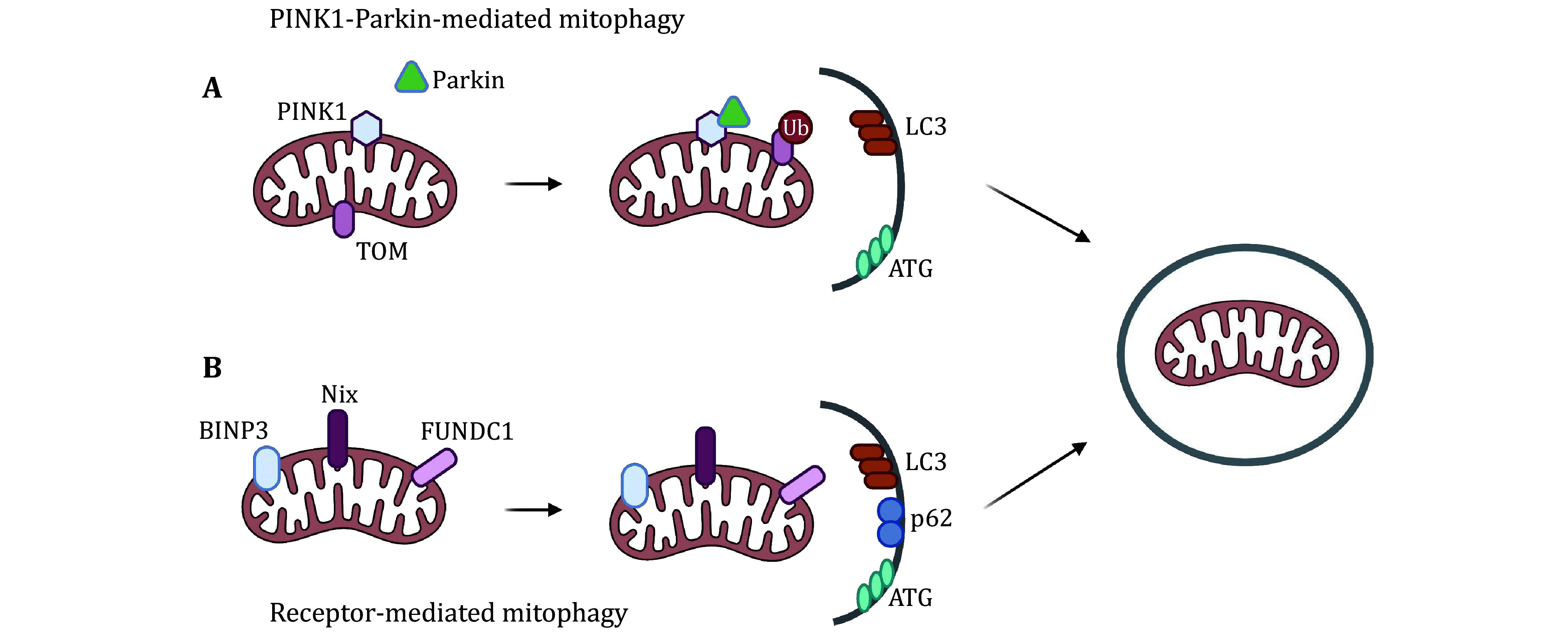
Mechanisms of mitophagy. **A** PINK1-Parkin-mediated mitophagy involves the activation of Parkin by PINK1, leading to the ubiquitination of mitochondrial proteins and recruitment of LC3 for mitochondrial degradation. **B** Receptor-mediated mitophagy utilizes mitochondrial membrane proteins like BNIP3, NIX, and FUNDC1, which bind LC3 directly via their LIR domains to target mitochondria for lysosomal degradation

PINK1/Parkin-mediated mitophagy is the best characterized mitophagy pathway, but other alternative pathways have been revealed in recent years: receptor mediated mitophagy. Some specific proteins on the mitochondrial membrane can be used as receptors for mitophagy, including BNIP3, NIX, FUNDC1, *etc.* (Bi *et al.* 2024; Lv *et al.* 2017; Sun *et al.* 2024). These membrane proteins can bind LC3 directly with their LIR domain and transport mitochondria to lysosomes for degradation (Fig. 1).

Mitophagy is intricately linked to the pathogenesis of a myriad of diseases, encompassing neurodegenerative disorders, cardiovascular diseases, cancers, and metabolic disorders (Ajoolabady *et al.* 2022; Aventaggiato *et al.* 2021; Malpartida *et al.* 2021; Yan *et al.* 2024). For instance, heightened mitophagy is correlated with various acute and chronic brain pathologies, and dysregulation in this process can result in the accumulation of nonfunctional mitochondria, potentially exacerbating cellular dysfunction and disease progression (Xia *et al.* 2023).

## MITOPHAGY AND MITOCHONDRIAL ROS

Mitochondria are the center of cellular energy metabolism. Under aerobic conditions, mitochondria produce a large amount of ATP through the TCA cycle and oxidative phosphorylation process to provide energy for cellular activities. The oxidative phosphorylation of mitochondria inevitably produces superoxide free radicals, which are the main source of ROS in cells. ROS are generally considered to be a by-product of mitochondrial redox (Adam-Vizi and Chinopoulos 2006). Overproduction of ROS can inflict damage on mitochondrial DNA (mtDNA), diminish the mitochondrial membrane potential, and induce the oxidation of proteins and lipids through oxidative stress (Srinivas *et al.* 2019). Lipid peroxidation stands as one of the most critical processes in ROS-mediated cell damage and death, and it is also a central event in the mediation of ferroptosis (Wang *et al.* 2023). Mitochondria are also the primary target of ROS. On one hand, mitochondria are the most important place for phosphatidylethanolamine (PE) synthesis in cells, and the content of PE in cell membrane is closely related to the sensitivity of ferroptosis, because PE-polyunsaturated fatty acid (PE-PUFA) is the main substrate of lipid peroxidation (Wang *et al.* 2023). On the other hand, cardiolipin, which only exists in mitochondria, is rich in polyunsaturated fatty acids and very vulnerable to oxidative attack. Mitophagy is a fundamental mitochondrial quality control mechanism that eliminates ROS and damaged mitochondria. Under the oxidative stress of ROS, some specific mitochondrial lipids such as cardiolipin and ceramide are critical for effective responses to mitophagy by binding LC3 to recruit phagophores to damaged mitochondria (Chu *et al.* 2023). The oxidation of certain proteins also triggers adaptive mitophagy, which helps cells survive oxidative damage.

Mitophagy, while typically serving as a protective mechanism to eliminate excessive ROS and mitochondrial damage, can be excessively and pathologically activated under conditions of overwhelming or prolonged mitochondrial dysfunction. In certain scenarios, the ensuing cell death is a direct consequence of mitophagy-dependent ROS generation. Consequently, mitophagy may augment ROS levels through a positive feedback loop, thereby exacerbating cellular damage.

## MITOPHAGY AND MITOCHONDRIAL IRON METABOLISM

Mitochondria also play a pivotal role in iron metabolism, a function that is crucial yet often underappreciated (Ru *et al.* 2024). Iron's fundamental role is to engage in redox reactions, which are vital for numerous enzymatic processes (Lennicke and Cocheme 2021; Yan *et al.* 2024). However, the very property that makes iron essential, its ability to transfer electrons, also renders it potentially hazardous. For example, iron can provide electrons to O_2_ and H_2_O_2_ through Fenton reaction to generate hydroxyl radical or hydroperoxide radical and superoxide anion, which become a risk for ferroptosis (Roemhild *et al.* 2021; Tang *et al.* 2021; Zhao 2023). Therefore, the acquisition, utilization and storage of iron are strictly regulated within cells. In eukaryotes, a significant portion, approximately 20%–50%, of the cellular iron is sequestered within mitochondria, either as mitochondrial ferritin (FTMT), heme, or iron-sulfur (Fe-S) clusters (Galy *et al.* 2024; Read *et al.* 2021). FTMT is predominantly expressed in tissues with high oxygen consumption, such as the central nervous system, where it is crucial for safeguarding mitochondria against ROS generated by iron (Ward and Cloonan 2019). This protection is vital, as many mitochondrial proteins require iron for their proper metallization, folding, and stability. Disruptions in mitochondrial iron homeostasis are implicated in the pathogenesis and progression of numerous diseases. Exploring the molecular mechanism regulating mitochondrial iron homeostasis will help to further understand the role of iron in mitochondrial function and human diseases.

Mitochondria are crucial in regulating iron metabolism, and disruptions in this process can adversely affect mitochondrial function (Ward and Cloonan 2019). To preserve metabolic and biological homeostasis, mitochondria are constantly undergoing fission and fusion, which are essential for maintaining their health and function. This dynamic process allows cells to segregate damaged mitochondria from those that are functioning properly and facilitates the selective removal of impaired mitochondria through mitophagy (Chen 2022; Li *et al.* 2022b). This mechanism is vital for protecting cells from the damage of free radicals and preventing cell death. The damaged mitochondria are transported to lysosomes for degradation, and in this process, the iron contained within the mitochondria can be released and recycled or stored, thus maintaining iron homeostasis within the cell.

## THE DEFENSE ROLE OF MITOPHAGY AGAINST FERROPTOSIS

The role of mitochondria in ferroptosis is a subject of debate, and similarly, mitophagy appears to exert opposing effects on the regulation of ferroptosis under various conditions (Liu *et al.* 2023). On one hand, mitophagy has been reported to negatively regulate ferroptosis by facilitating the removal of damaged mitochondria and excessive ROS under certain circumstances (Chen *et al.* 2024; Ren *et al.* 2024). As the cellular energy center, mitochondria are in constant turnover for quality control. Through mitophagy, cells manage to remove the senescent and damaged mitochondria, which are prone to produce more ROS. Therefore, mitophagy is thought to protect the cells against ferroptosis by diminishing oxidative damage, as evidenced in these studies (Table 1).

**Table 1:** Mitophagy prevents ferroptosis

Protein/ Molecule	Function	Effect on mitophagy	Effect on ferroptosis	Reference
HIF	Transcription factor; Regulating the expression of mitophagy-inducing factors	↑	↓	Li *et al.* 2022b
CISD3	Mitochondrial iron homeostasis	–	↓	Li *et al.* 2024a
Nrf2	Transcription factor; Regulating the expression of PINK1 and p62	↑	↓	Chen 2022
CCCP	Mitophagy inducer	↑	↓	Jiang *et al.* 2024
FUNDC1	Mitophagy receptor	↑	↓	Pei *et al.* 2021
Silibinin	Antihepatotoxic	↑	↓	Song *et al.* 2022
Olaparib	PARP inhibitor	↑	↓	Liu *et al.* 2024
Nlrp6	Multifaced innate immune sensor	↑	↓	Shen *et al.* 2024
NETs	Capturing and eliminating infectious microorganisms	↓	↑	Chu *et al.* 2023

HIF can promote mitophagy and protect cells against ferroptosis. Previous studies have demonstrated that certain pathways activating HIF signaling provide robust protection against ferroptosis by enhancing mitophagy. Enhanced mitophagy results in diminished mitochondrial oxidative stress and damage. HIF, recognized as a pivotal transcription factor in response to hypoxia and mediating cellular adaptation, promotes mitophagy by regulating key mitophagy-inducing factors such as BNIP3, PINK1/Parkin, and heme oxygenase 1 (HO-1, HMOX1). Under these conditions, mitophagy may contribute to the defense against ferroptosis by reducing ROS production and limiting mitochondrial damage. PPARγ activation inhibits ferroptosis in chondrocytes through the PINK1/Parkin-dependent mitophagy pathway. Peroxisome proliferator-activated receptor-γ (PPARγ) is a ligand-activated transcription factor. It is a key regulator in maintaining cartilage health and has a chondroprotective role in osteoarthritis (OA). To explore the effect of PPARγ on ferroptosis in OA and its potential mechanisms, Xue *et al*. ultimately demonstrated that PPARγ activation inhibits chondrocyte ferroptosis in OA and that this chondroprotective effect is associated with the activation of PINK1/Parkin-dependent mitophagy.

Activation of mitophagy can rescue the CISD3 knockdown-induced ferroptosis. CISD3 is a member of the NEET protein family. It is reported to exert a regulatory role in cancer progression and ferroptosis. Recently, Li *et al*. found that CISD3 knockdown significantly accelerated lipid peroxidation and accentuated free iron accumulation, leading to promoted ferroptosis. They also found that genetic and pharmacological activation of mitophagy could rescue the CISD3 knockdown-induced ferroptosis by eliminating the damaged mitochondria.

Nrf2 promotes mitophagy and confers resistance against ferroptosis. Nrf2 is a transcription factor that can be activated under oxidative stress and activates following tissue injury. Experimental evidence supports the role of Nrf2 in mitophagy by up-regulating the expression of PINK1 and p62. Nrf2 is also proven to inhibit ferroptosis by removing oxidant generation and mitochondrial damage.

Mitophagy induced by CCCP can completely block ferroptosis. To investigate the role of mitochondria in ferroptosis, researchers from Jiang’s group found that CCCP, a commonly used mitophagy inducer, could completely abolish lipid ROS accumulation and cysteine-deprivation-induced ferroptosis.

FUNDC1-dependent mitophagy promotes ACSL4-mediated ferroptosis. Pei *et al*. illustrated that FUNDC1 ablation unmasked HFD-evoked rises in fatty acid synthase ACSL4, mitochondrial O_2_ production, mitochondrial injury, as well as a decrease in mitophagy, leading to increased ferroptosis.

Silibinin inhibits ethanol- or acetaldehyde-induced ferroptosis. Song *et al*. found that ethanol and acetaldehyde promoted ferroptosis by promoting the autophagic degradation of ferritin and reducing mitophagy which retards ROS-induced lipid peroxidation and ferroptosis, which could be rescued by silibinin.

Low-dose Olaparib improves septic cardiac function by reducing ferroptosis via accelerated mitophagy flux. Olaparib is a PARP inhibitor. In a recent study, Liu *et al*. found that low-dose Olaparib effectively targeted and mitigated markers associated with ferroptosis. Moreover, low-dose Olaparib accelerated mitophagy flux, promoted the clearance of damaged mitochondria post-sepsis and improved mitochondrial respiration quality. The mechanism through which Olaparib inhibits ferroptosis and reduces the inflammatory response is attributed to its facilitation of mitophagy, thus favoring mitochondrial integrity.

Nlrp6 protects from corticosterone-induced NSPC ferroptosis by modulating RIG-1/MAVS-mediated mitophagy. Nlrp6 is an innate immune sensor. Recently, Shen *et al*. have demonstrated that Nlrp6 deficiency caused a suppression of RIG-1/MAVS-mediated mitophagy that drove NSPC ferroptotic death by impairing the system Xc−/GSH/GPX4 axis. Furthermore, it has been found that SCFAs could upregulate Nlrp6 expression and promote RIG-1/MAVS-mediated mitophagy to prevent corticosterone-induced NSPC ferroptosis.

NETs drive intestinal microvascular endothelial ferroptosis by impairing FUNDC1-dependent mitophagy. NETs are capable of capturing and eliminating infectious microorganisms. The present study demonstrated that the formation of NETs hindered mitophagy levels and exacerbated mitochondrial dysfunction. Moreover, Chu *et al*. revealed that the mitophagy process, dependent on FUNDC1, significantly promoted ferroptosis in the intestinal endothelium, thereby leading to microvascular dysfunction. Therefore, NETs can initiate endothelial ferroptosis by impeding the activation of FUNDC1-requiring mitophagy.

## THE PRO-FERROPTOTIC ROLE OF MITOPHAGY

Despite compelling evidence to support the defense role of mitophagy against ferroptosis, recent studies also reveal a pro-ferroptotic role of mitophagy (Javadov 2022). A large amount of iron is stored and utilized in mitochondria and will be released to the cytosol through mitophagy (Yamashita *et al.* 2024; Zhou *et al.* 2024). Under some severe damages characterized by excessive mitophagy, overwhelming iron release promotes ferroptosis significantly (Zhou *et al.* 2024). Furthermore, mitophagy may result in the production of excessive ROS during the degradation of mitochondria, which also makes mitophagy a facilitator of ferroptosis (Table 2).

Dynamic *O*-GlcNAcylation coordinates ferritinophagy and mitophagy to activate ferroptosis. Yu *et al*. observed a biphasic change of protein *O*-GlcNAcylation that modulates ferroptosis. Pharmacological or genetic inhibition of *O*-GlcNAcylation promoted ferritinophagy and mitophagy, supplying an additional source of labile iron and rendering the cell more sensitive to ferroptosis. To be noticed, they also found that mitochondria acted as a buffering pool of labile iron. Under ferroptotic stress, the labile iron might be transported to mitochondria as a defense strategy against ferroptosis. Conversely, promoted mitophagy releases the iron stored in mitochondria, leading to elevated levels of free cellular iron and the sensitivity to ferroptosis.

**Table 2:** Mitophagy promotes ferroptosis

Protein/ Molecule	Function	Effect on mitophagy	Effect on ferroptosis	Reference
OGT	Post-translational modification	OGT↓ Mitophagy↑	OGT↓ Ferroptosis↑	Yu *et al.* 2022
Aβ1-40	β-amyloid protein in Alzheimer’s disease	Aβ1-40↑ Mitophagy↑	Aβ1-40↑ Ferroptosis↑	Li *et al.* 2022a; Song *et al.* 2024
Sevoflurane	Anesthetic; Antitumor	Sevoflurane↑ Mitophagy↑	Sevoflurane↑ Ferroptosis↑	Ren *et al.* 2020; Xu *et al.* 2022
FUNDC1	Mitophagy receptor	FUNDC1↑ Mitophagy↑	FUNDC1↑ Ferroptosis↑	Bi *et al.* 2024; Zhu *et al.* 2023
Myoferlin	Oncoprotein	Myoferlin↑ Mitophagy↑	Myoferlin↑ Ferroptosis↑	Rademaker *et al.* 2022
ATG5	Mitophagy receptor	ATG5↓ Mitophagy↓	ATG5↓ Ferroptosis↓	Basit *et al.* 2017; Nishida *et al.* 2009
PINK1	Mitophagy	PINK1↓ Mitophagy↓	PINK1↓ Ferroptosis↓	Basit *et al.* 2017; Lv *et al.* 2016
Drp1	Mitochondria fission	Drp1↓ Mitophagy↓	Drp1↓ Ferroptosis↓	Basit *et al.* 2017
SIRT3	Mitophagy	SIRT3↑ Mitophagy↑	SIRT3↑ Ferroptosis↑	Li *et al.* 2024b
HO-1	Heme-degrading enzymes	HO-1↑ Mitophagy↑	HO-1↑ Ferroptosis↑	Li *et al.* 2024a
Mdivi-1	Mitochondrial inhibitors	Mdivi-1↓ Mitophagy↓	Mdivi-1↓ Ferroptosis↓	Yang *et al.* 2024
NKAα1	Na^+^/K^+^-ATPase	NKAα1↓ Mitophagy↓	NKAα1↓ Ferroptosis↓	Zhang *et al.* 2024a

β-amyloid protein induces mitophagy-dependent ferroptosis. Li *et al*. reported that Aβ1-40 initiates ferroptosis in pericytes through a mitophagy-dependent mechanism involving the CD36/PINK1/Parkin pathway. This peptide was found to induce mitochondrial damage and enhance mitophagy, which in turn led to elevated levels of iron ions and lipid reactive oxygen species (ROS), thereby promoting ferroptosis.

Sevoflurane induces ferroptosis by activating the ATF4-CHAC1 pathway. Sevoflurane, a volatile anesthetic, is commonly used in clinical operations. It has been recently reported to exert antitumor physiologic effects in several tumors. It was found to increase ROS levels and Fe^2+^ concentration by activating the ATF4-CHAC1-dependent mitophagy.

Ablation of FUNDC1-dependent mitophagy renders myocardium resistant to paraquat-induced ferroptosis. Interestingly, a totally opposite role of FUNDC1 was reported by researchers from Ren’s group. They revealed that paraquat challenge overtly upregulated levels of FUNDC1 and ferroptosis, while ablation of FUNDC1 inhibited paraquat-induced increase in BODIPY lipid peroxidation and mitigated the effects against mitophagy and ferroptosis.

Myoferlin targeting triggers mitophagy and primes ferroptosis. Myoferlin is a recently identified oncoprotein. Pharmacological inhibition of Myoferlin triggered mitophagy and ROS accumulation culminating with lipid peroxidation and ferroptosis. Additionally, the mitophagy inhibitor Mdivi1 and iron chelators are capable of inhibiting Myoferlin-related ROS production and ferroptosis.

Mitochondrial complex I inhibition triggers a mitophagy-dependent ROS increase leading to ferroptosis. Basit *et al*. demonstrated that inhibition of complex I of the mitochondrial respiratory chain induces mitophagy-dependent ROS accumulation, culminating in ferroptosis. They found that knockdown of ATG5 or PINK1 abrogated mitophagy induction, cellular ROS elevation, and ferroptosis, whereas Drp1 knockdown promoted mitochondrial filamentation and mitigated ferroptosis. It is plausible that mitophagy could lead to the production of increased ROS during the degradation of mitochondria, but the precise mechanism by which autophagy/mitophagy increases ROS levels warrants further investigation.

Inhibition of SIRT3 enhances the sensitivity of glioblastoma cells to RSL3-induced ferroptosis by promoting mitophagy and suppressing SLC7A11. The accumulation of mitochondrial iron and reactive oxygen species (ROS) triggered by SIRT3 inhibition initiates mitophagy. Concurrently, the downregulation of SLC7A11 expression reduces cystine uptake, thereby lowering glutathione (GSH) levels, and making the cells more susceptible to ferroptosis.

Heme oxygenase-1 (HO-1) is an enzyme involved in heme degradation, recently Li *et al*. found that its upregulation promotes the breakdown of heme, resulting in an increased release of intracellular iron ions. This elevation in iron ions may trigger the activation of mitophagy, as the accumulation of iron ions within the mitochondria can lead to mitochondrial damage, thereby activating mitophagy. This process provides an additional source of iron, further promoting lipid peroxidation and ferroptosis.

Mdivi-1 inhibits excessive mitophagy and modulates ferroptosis proteins: as a mitochondrial autophagy inhibitor, Yang *et al*. discovered that Mdivi-1 reduces the flux of mitophagy in HT22 cells by restricting mitochondrial fission. Concurrently, it elevates the expression of GPX4 and FTH1 proteins while decreasing the expression of ACSL4 protein; these alterations in protein expression collectively promote ferroptosis in cells.

NKAα1 is a Na^+^/K^+^-ATPase, and its deficiency inhibits mitophagy, affecting the initiation of Parkin-mediated mitophagy. As Zhang *et al*. Reported, NKAα1 forms a complex with SLC7A11, promoting its function in resisting ferroptosis and maintaining the balance of intracellular iron metabolism and redox status.

## CONCLUDING REMARKS

Over the past few years, great strides have been made in illustrating the mechanism of ferroptosis. Cellular metabolism and ferroptosis closely interact with one another. As the center of cell metabolism, mitochondrion plays a critical role in iron metabolism and cell death, which makes it a focal point in the exploration of ferroptosis. However, after years of investigation, the relationship between mitochondria and ferroptosis remains to be further elucidated, given the highly controversial findings. Although it has been found that a series of metabolic activities in mitochondria could facilitate ferroptosis, there are also anti-ferroptosis defense systems present in mitochondria. Therefore, it will be challenging to attribute a universal function, either pro-ferroptotic or anti-ferroptotic, to mitochondria as a whole.

**Figure 2:**
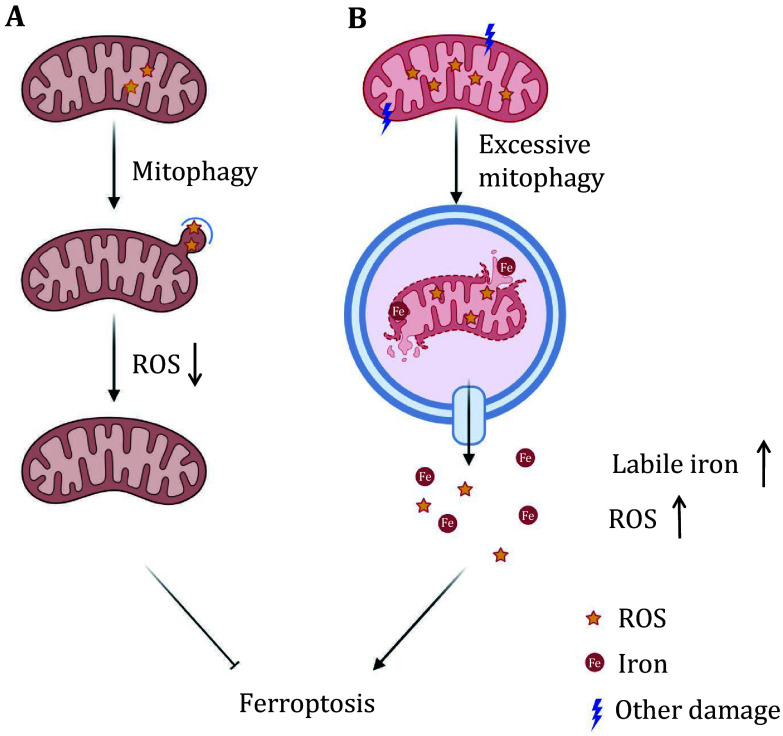
The complex role of mitophagy in ferroptosis. **A** Mitophagy may inhibit ferroptosis by removing dysfunctional mitochondria and thereby reducing ROS levels. **B** Excessive mitophagy could promote ferroptosis by releasing iron, increasing the levels of labile iron, and sensitizing cells to ferroptosis. Mitophagy plays a dual role in ferroptosis, potentially protecting cells or promoting ferroptosis depending on the extent of mitochondrial damage and the release of iron

Similarly, it seems that mitophagy also has a controversial role in ferroptosis. In this review, we analyzed some of the conflicting data in the literature regarding the role of mitophagy in ferroptosis. In the author’s view, mitophagy could either promote or inhibit ferroptosis depending on the two major functions of mitochondria. On one hand, the activation of mitophagy is protective in some contexts by removing dysfunctional mitochondria and decreasing oxidative damage. Mitochondria are a major source of ROS, which could be eliminated by mitophagy. Accumulated ROS would increase the risk of ferroptosis and under such circumstances, mitophagy could help to repress ferroptosis. Notably, the relationship between ROS levels and mitophagy is actually more complicated. In some cases, overwhelming mitochondrial damage can induce excessive, pathological activation of mitophagy, and mitophagy could increase ROS levels through a positive feedback loop. On the other hand, mitochondrion is a large storage pool of iron. Iron overload is a distinguishing characteristic of ferroptosis and is required for the formation of lipid peroxides. When mitophagy happens, mitochondria are delivered to lysosomes and degraded into small molecules. In this process, the iron stored in mitochondria might be released into the cytosol, increasing the level of labile iron and rendering the cell more sensitive to ferroptosis. In this case, mitophagy might play a pro-ferroptotic role (Fig. 2). Recently, the important role of mitochondria as the “buffering pool” for iron was illustrated by Prof. Chen’s group. They found that under ferroptotic stress, the level of mitochondrial iron increased dramatically, suggesting that mitochondria may serve as a buffering organelle that sequesters labile iron to prevent ferroptosis. However, subsequently activated mitophagy may release the iron in mitochondria to cytosol and provide an additional source of iron for lipid peroxidation.

In conclusion, mitophagy plays a dual role in ferroptosis. In relatively milder cases, appropriate mitophagy removes accumulated ROS and represses ferroptosis. In contrast, excessive mitophagy, which usually happens under more severe mitochondrial damages, may lead to more ROS production and mitochondrial iron release, and therefore promotes ferroptosis. This is just one possible explanation for the controversial behavior of mitophagy in ferroptosis, further studies are still needed to clarify the detailed mechanisms to solve this contradiction.
